# Inflammatory pathways in the mechanism of parturition

**DOI:** 10.1186/1471-2393-7-S1-S7

**Published:** 2007-06-01

**Authors:** Jane E Norman, Shrikant Bollapragada, Mei Yuan, Scott M Nelson

**Affiliations:** 1 University of Glasgow Division of Developmental Medicine, Glasgow Royal Infirmary, Queen Elizabeth Building, 10 Alexandra Parade, Glasgow, G31 2ER, UK

## Abstract

Increasing evidence suggests that parturition is an inflammatory process. In this brief overview, inflammatory events occurring in association with parturition, and the mechanism by which they may contribute to labour and delivery will be discussed. Mention will be made of how this information may be of use in regulating the timing and the onset of parturition.

## Background

Preterm birth is the single biggest cause of perinatal mortality and morbidity. Preterm birth rates are rising in all developed countries. In Scotland, there has been a progressive rise in preterm birth rates from 5.8% in 1980 to 7.6% in 2005 [1]. Work from our own and other groups increasingly suggests that parturition is an inflammatory process, and we anticipate that further understanding of these events will contribute to interventions to prevent preterm birth. Inflammation can either be an acute or a chronic event. The acute event is characterised by its short duration (minutes – days), blood vessel dilation and leakiness, exudation of fluid and plasma proteins and emigration of leukocytes.

## Myometrial events

Our interest in the possible role of inflammation in preterm labour was sparked in 1999. We initially performed myometrial biopsies from a group of women during Caesarean section in labour (n = 18) being careful to exclude women with clinical signs of infection, those who had received oxytocic or prostaglandin drugs and those who had a cervical dilation of less than 4 cm or greater than 8 cm. We also obtained myometrial biopsies from a cohort of women who underwent Caesarean section prior to the onset of labour. Histology slides were prepared and stained with relevant antibodies: neutrophil elastase or CD15 to identify neutrophils, CD68 to identify macrophages, CD3 for T cells and CD20 for B cells. Slides were then examined, and the number of cells counted in five high powered fields by two observers working independently and who were unaware of the clinical details of the subject. We demonstrated that there was a massive influx of inflammatory cells (neutrophils and macrophages) into both the lower and the upper segment of myometrium in association with physiological labour [2]. The magnitude of leukocyte invasion was greatest in the lower segment, with an increase in T cell density only occurring in the lower segment, and no increase in B cell density in either location.

Later, by performing Northern analysis on biopsies of myometrium taken from each of labouring and non labouring women, we established that this leukocytic influx was paralleled by up-regulation of pro-inflammatory cytokine mRNA expression, specifically IL-1β, IL-6 and IL-8 [3]. In subsequent studies where we stained back to back sections with antibodies against the cytokines IL-1β, IL-6 and IL-8 and with leukocyte cell surface markers we determined that protein expression of these pro-inflammatory cytokines was largely (although not exclusively) confined to invading leukocytes [4]. Lastly, we examined ICAM-1, an endothelial adhesion molecule which facilitates adhesion, arrest and transmigration of leukocytes and demonstrated that myometrium expresses greater amounts of ICAM-1 during labour, compared to immediately prior to parturition [5]. Collectively this body of work in conjunction with studies by other groups suggests a co-ordinated mechanism by which leukocytes can traffic into the myometrium, and can release pro-inflammatory cytokines to amplify or initiate the process of parturition.

Our data do not prove that pro-inflammatory cytokines are integral to the pathophysiology of labour. However, there is good evidence that pro-inflammatory cytokines will contribute to stimulation of uterine contractions. For example, in cultured human myometrial cells, Tribe et al showed that IL-1β induces both basal and store operated calcium entry in myometrial smooth muscle cells, thus directly increasing their contractile ability/activity [6]. Oger and colleagues demonstrated that IL-1β, stimulates phosphodiesterase (PDE) activity [7]. Since PDE breaks down cAMP, which maintains myometrial quiescence, IL-1β would thus stimulate uterine contractions. Others have shown that IL-1βB stimulates production of both PGF2α production [8] and PGF2α receptors, which will further contribute to myometrial contractions. Further work from other groups also established that pro-inflammatory cytokines may contribute to the myometrial remodelling which occurs with labour [9]. These data all need to be interpreted with caution, because the experiments were performed in myometrial cells rather than whole tissue or in vivo, but if they can be replicated in more physiological conditions it seems likely that the influx of leukocytes (which is the hallmark of inflammation) can contribute directly to the pathophysiology of parturition.

A more recent study in our department using DNA microarrays to identify differences in the transcriptional profiles in labouring and non labouring myometrium, demonstrated that out of the top 5 upregulated genes, two are primarily related to inflammation (interleukin 8 and chemokine [C-X-C motif] ligand 5), and two are related to tissue remodelling (matrix metallopeptidases 1 and 10 respectively) (Bollopragada et al, unpublished data). We are currently analysing this data in more detail, however, consistent with our previous work it indicates the importance of inflammation in the process of parturition.

## Cervical events

The description of cervical ripening as an inflammatory reaction is not new, being made by Liggins in the early 1980s. Again, we have shown that labour is associated with a massive influx of leukocytes into the cervix [3], and that pro-inflammatory cytokines and cell adhesion molecules are co-incidentally expressed. Additionally, nitric oxide, made from L-arginine by one of three nitric oxide synthase enzymes, seems to have an important role in cervical ripening. Each of the three nitric oxide synthase enzymes is produced in the cervix, and we and others have shown increased expression of inducible NOS in association with cervical ripening [10,11]. Nitric oxide release and the concurrent up-regulation of inducible nitric oxide synthase are common events in inflammation, and suggest that these pro-inflammatory agents may be useful in the initiation of parturition. Consequently we have been investigating clinically the use of nitric oxide donors as cervical ripening agents. Nitric oxide donors have a significant advantage over prostaglandins, the current agent of choice for cervical ripening, because nitric oxide donors relax the uterus. Uterine contraction before the cervix is ripe is ineffective in progressing labour, and merely reduces blood flow to the baby. In 5 – 10% women this is sufficient to cause fetal distress (which may require treatment by Caesarean section), and consequently, cervical ripening is carried out as an inpatient procedure. Although less effective than prostaglandins in the doses currently investigated, nitric oxide donors appear to be safe and well tolerated for cervical ripening, and tend to be preferred by women [12-14]. They may therefore be suitable for use on an outpatient basis, and their efficacy in this regard is currently under investigation [15].

## Fetal membranes

In contrast to data from the cervix and myometrium, we have been unable to show an increase in leukocyte cell density in fetal membranes, comparing labouring with non labouring tissue [16]. There is however a significant upregulation of IL-1β, and IL-6 in labour. A genomic study from another group has confirmed that fetal membranes in labour do indeed have an acute inflammatory signature [17]. The source of this inflammatory activity is unknown: whether production of cytokines from resident inflammatory cells increases, or whether other cells contribute to the increase in IL-1β and IL-6 production. Further studies are required to investigate this issue, however, comparative analysis of this membrane genomic data with cervical and myometrial gene array data derived from our own group, clearly identifies several genes which are up-regulated in all three tissue types, with pro-inflammatory and chemotactic genes feature prominently, suggesting that labour is associated with a core inflammatory response in all gestational tissues.

## Peripheral blood

Surprisingly little attention has been given to activity and gene expression of leukocytes in peripheral blood at the time of parturition. We have recently embarked on studies to address this issue. Preliminary studies have shown that chemotaxis of peripheral blood leukocytes and reactive oxygen species production is greater in blood samples from labouring compared with non labouring women at term. If these data are confirmed in larger studies it suggests that leukocytes may be primed and activated in the peripheral circulation at the onset of labour, with subsequent invasion of the myometrium and cervix, thereby potentially initiating and propagating cervical ripening and uterine contractions. Therefore circulating leukocytes are an attractive potential therapeutic target – with inactivation of peripheral leukocytes preventing their initial influx into myometrium and cervix and their subsequent cytokine mediated stimulation of uterotonic agents.

## Conclusion

Evidence that labour at term is an inflammatory reaction continues to accumulate. Inflammatory events can be observed in each of the myometrium, cervix, fetal membranes and peripheral blood. The inflammatory events which we and others have demonstrated during parturition at term are paralleled by the effects of infection in preterm parturition. It is to be hoped that further understanding of these events will lead to novel therapeutic strategies for the treatment and prevention of preterm labour in due course, and to improved interventions for cervical ripening and induction of labour.

## Competing interests

JEN received consultancy fees from Glaxo Smith Kline in 2005. JEN and SN receive funding from charitable and governmental bodies to conduct research in parturition. We declare that we have no other conflicts of interest.

## Authors' contributions

JEN wrote the manuscript. SMN, SB and MY contributed data and commented on the final version of the manuscript.
